# Serum polychlorinated biphenyl levels and circulating miRNAs in non-obese women with and without polycystic ovary syndrome

**DOI:** 10.3389/fendo.2023.1233484

**Published:** 2023-09-18

**Authors:** Edwina Brennan, Alexandra E. Butler, Daniel S. Drage, Thozhukat Sathyapalan, Stephen L. Atkin

**Affiliations:** ^1^ School of Medicine, Royal College of Surgeons in Ireland-Medical University of Bahrain, Busaiteen, Bahrain; ^2^ School of Geography, Earth and Environmental Sciences, University of Birmingham, Birmingham, United Kingdom; ^3^ Queensland Alliance for Environmental Health Sciences, The University of Queensland, Brisbane, QLD, Australia; ^4^ Hull York Medical School, University of Hull, Hull, United Kingdom

**Keywords:** microRNA (miRNA), polychlorinated biphenyls (PCBs), polycystic ovary syndrome (PCOS), endocrine disruptors, organic pollutants

## Abstract

**Introduction:**

Polychlorinated biphenyls (PCBs), organic lipophilic pollutants that accumulate through diet and increase with age, have been associated with polycystic ovary syndrome (PCOS) and shown to affect microRNA (miRNA) expression. This work aimed to determine if PCBs were associated with circulating miRNAs and whether there were any correlations with serum PCB/miRNA levels and hormonal changes.

**Methods:**

29 non-obese PCOS and 29 healthy control women, with similar age and body mass index (BMI), had their serum miRNAs measured together with 7 indicator PCBs (PCB28, PCB52, PCB101, PCB118, PCB138, PCB153, PCB180) using high resolution gas chromatography coupled with high resolution mass spectrometry.

**Results:**

In the combined study cohort, four miRNAs (hsa-miR-139-5p, hsa-miR-424-5p, hsa-miR-195-5p, hsa-miR-335-5p) correlated with PCBs, but none correlated with metabolic parameters. hsa-miR-335-5p correlated with FSH. When stratified, 25 miRNAs correlated with PCBs in controls compared to only one (hsa-miR-193a-5p) in PCOS; none of these miRNAs correlated with the metabolic parameters of BMI, insulin resistance, or inflammation (C-reactive protein, CRP). However, of these 25 miRNAs in controls, hsa-miR-26a-5p, hsa-miR-193a-5p, hsa-miR-2110 and hsa-miR-195-5p positively correlated with luteinizing hormone (LH), hsa-miR-99b-5p and hsa-miR-146b-5p correlated with estradiol, hsa-miR-193a-5p correlated with progesterone, hsa-miR-195-5p correlated with follicle stimulating hormone (FSH), and hsa-miR-139-5p and hsa-miR-146b-5p negatively correlated with anti-müllerian hormone (AMH) (all *p*<0.05). hsa-miR-193a-5p in PCOS cases correlated with estradiol.

**Conclusion:**

In this cohort of women, with no difference in age and BMI, and with similar PCB levels, the miRNAs correlating to PCBs associated with menstrual cycle factors in healthy menstruating controls versus the anovulatory PCOS subjects. The PCB-associated miRNAs did not correlate with non-reproductive hormonal and metabolic parameters. This suggests that PCB effects on miRNAs may result in changes to the hypothalamo-ovarian axis that may thus affect fertility.

## 1 Introduction

Polychlorinated biphenyls (PCBs) are synthetic chemicals that were once widely manufactured from 1929 for their use in electrical equipment as dielectric and hydraulic fluids (1). Structurally, PCBs consist of a biphenyl ring chemically bound with 1 – 10 chlorine atoms with 209 possible congeners due to the number and position of the chlorine substituents which has implications for exposure, toxicity, and environmental fate. Highly chlorinated PCBs (≥5 chlorine atoms) have low volatility, high lipophilicity, and low biotransformation rates compared to low chlorinated PCBs (≤ 4 chlorine atoms) (2). Twelve PCB congeners, including PCB118, are co-planar (lack both ortho chlorine substituents) and are termed dioxin-like PCBs due to their ability to bind to the aryl hydrocarbon receptor (AhR). Estimated half-lives of indicator PCBs, those most common in commercial mixture and the environment, range from 2.4 – 5.5 years for PCB28 (trichloro) to 11.5 years for PCB180 (heptachloro) (3, 4). Exposure to highly chlorinated PCBs generally results from dietary consumption due their bioaccumulation in the food chain (5) while inhalation is the main route of exposure of low chlorinated PCBs (6).

Due to the growing concerns on human health, the manufacture of PCBs was banned in 1979 followed by their inclusion into the Stockholm Convention of Persistent Organic Pollutants (7) and international ban in 2004. Although banned, leakage from products in landfill (8) and the release of unintentional PCB by-products from pigment manufacturing (9), result in continued exposure. More recently, the EU reduced the maximum allowable levels of PCBs in foodstuff (10). Daily background exposure estimates for total PCB exposure is estimated as 3.4 mg/kg for adults with 88% contribution from diet and 11% from inhalation (11). However, these estimates are based on studies that focused on indicator PCBs used to predict total PCB exposure, which may lead to significant under predictions (12). PCBs are classified as endocrine disruptors due to their observed thyroidogenic, estrogenic, and antiandrogenic action (13) and are reported to affect the epigenome (14).

Guida et al. (15) published the first study reporting molecular evidence of PCB effects on microRNA (miRNA) expression in humans where they found that PCB169 correlated with miRNA-191 expression in pregnant women who underwent therapeutic abortion due to fetal deformation. MiRNAs are short non-coding endogenous RNA transcripts of ~22 nucleotides in length that repress gene expression post-transcriptionally through complementary binding to target messenger RNA (mRNA) (16). Most miRNAs bind to the 3’ untranslated regions of target mRNAs, resulting in either inhibition of target translation or promotion of target degradation (17). Given the short nucleotide sequence, a single miRNA may be complementary with hundreds of mRNA 3’ untranslated regions and a single mRNA may be influenced by many miRNAs (18). As miRNAs mostly target protein-coding transcripts, they are involved in nearly all networks that regulate developmental and pathological processes. Therefore, it is not surprising that altered miRNA expression has been reported in PCOS (19–22).

Polycystic ovary syndrome (PCOS) is associated with menstrual dysfunction, infertility, hirsutism, acne, obesity and metabolic syndrome (23). Among women of reproductive age, PCOS has a reported prevalence of 6-10% (24). PCOS is a proinflammatory state with elevated inflammatory marker C-reactive protein (CRP) (25) and is a complex multigenetic heterogenous disorder with evidence of epigenetic and environmental influences resulting in varied phenotypes, clinical manifestations, and metabolic consequences (26).

Major issues in PCOS research are that obesity, insulin resistance and chronic inflammation are highly correlated to PCOS; therefore, statistical adjustment for these factors will over-adjust the PCOS effects. To account for this, a comparison between healthy controls and PCOS cohorts who were non-obese, non-insulin resistant, and without systemic inflammation was undertaken to determine if PCBs were associated with miRNAs, that would suggest endocrine disrupting effects may be effected through epigenetic alterations. Furthermore, we sought to determine whether there were any correlations with serum PCB/miRNA levels and hormonal changes.

## 2 Materials and methods

### 2.1 Study design

The study design was a case-control study. Participants were sequentially recruited in 2015 from the Hull *In Vitro* Fertilization (IVF) Unit, UK, following ethical approval from The Yorkshire and The Humber NRES ethical committee, UK (approval number 02/03/043). All study subjects were ethnically Caucasian. PCOS inclusion criteria included PCOS diagnosed using the revised 2003 Rotterdam criteria that requires 2 of 3 criteria to be met (27); clinical plus biochemical hyperandrogenism (indicated by a Ferriman-Gallwey score of 8 or greater; a free androgen index (FAI) of 4 or greater, a total testosterone level of 1.5 nmol/L or greater), oligomenorrhea or amenorrhoea together with polycystic ovaries as assessed by transvaginal ultrasound (TVUS). The following endocrine conditions were ruled out by performing appropriate testing: nonclassical 21-hydroxylase deficiency, hyperprolactinemia, Cushing’s disease and androgen-secreting tumors. Study participants had no other condition or illness and were otherwise deemed healthy. PCOS exclusion criteria included biochemical insulin resistance, obesity and a raised CRP indicative of chronic inflammation. Healthy control inclusion criteria included regular menstrual cycle, the cause of their infertility was either male factor infertility or unexplained infertility, and none had evidence of clinical/biochemical hyperandrogenism or polycystic ovaries by TVUS. Exclusion criteria for both healthy controls and PCOS were known immunological disease, diabetes, renal or liver insufficiency, acute or chronic infections, inflammatory disease, age <20 or >45 years, body mass index (BMI) >30 kg/m^2^, taking prescription or over the counter medication for nine months preceding the study. Subjects who fulfilled the inclusion and exclusion criteria were recruited prospectively for the single blood test that was taken at the same time as routine venesection as is shown in the flow chart (Supplementary **Figure S1**). Of the 58 participants recruited (29 PCOS and 29 healthy Controls), written informed consent was obtained from all (28).

### 2.2 Sample collection

At 21 days prior to IVF treatment when no hormonal treatment had been initiated, fasting blood samples were taken, centrifuged at 3500×g for 15 min at 4°C and stored at −80°C. Fasting blood glucose (FBG) was measured using a Synchron LX20 analyzer (Beckman-Coulter). Serum insulin was measured by competitive chemiluminescent immunoassay (DPC Immulite 2000 analyzer, Euro/DPC, Llanberis, UK). Homeostatic model assessment for insulin resistance (HOMA-IR) was calculated using the formula ((Insulin x glucose)/22.5) (29). CRP, total cholesterol (TC) and triglycerides (TG) were measured enzymatically (Synchon LX20 analyzer, Beckman-Coulter). Total serum lipid (TSL) was determined using the formula ((2.27 x TC) + TG + 62.3 mg/dL) (30). Anti-müllerian hormone (AMH) was measured using an immunoenzymatic assay (Beckman-Coulter). Estradiol, progesterone, luteinizing hormone (LH) and follicle stimulating hormone (FSH) were assayed on an Abbott Architect i4000 immunoassay analyzer (Abbott Diagnostics Division, UK). Glycosylated haemoglobin A1c (HbA1c) was measured using ion-exchange chromatography. Testosterone were measured by liquid chromatography tandem mass spectrometry (LC/MS/MS; Acquity UPLC-Quattro Premier XE-MS, Waters, Manchester, UK). An immunometric assay with fluorescence detection (DPC Immulite 2000 analyzer; upper limit 2.0 nmol/L) was used to measure sex hormone binding globulin (SHBG). The formula ((testosterone/SHBG) x 100) was used to calculate free androgen index (FAI).

### 2.3 MiRNA profiling and analysis

MiRNA analysis in serum samples was carried out as previously described (21). Briefly, total RNA was isolated from 200 µL serum aliquots using the miRCURY RNA Isolation Kit - Biofluids (Exiqon) following manufacturer recommended instructions with UniSp2, 4 and 5 (Exiqon) spike-ins used to assess RNA quality. Reverse transcription was performed on 4 µL of RNA in a reaction volume of 20 µL using Exiqon Universal cDNA Synthesis Kit II with UniSP6 and cel-miR-39-3p cDNA (Exiqon) used to assess efficiency. qPCR was run in Exiqon Serum/Plasma Focus microRNA PCR Panel, 384 well (V4.M) using QuantStudio 12K Flex Real-Time PCR System (ThermoFisher Scientific). Raw data was normalized with spike-in UniSp3. A no-template negative with a setting of DCt of 1 was used to eliminate false positives. A cut-off of DCt >7 between hsa-miR-23a-3p and hsa-miR-451a was used for haemolysed samples. Data were normalized against the global mean of all expressed miRNAs (Ct < 35) using GenEx qPCR analysis software (MultiD V6).

### 2.4 Polychlorinated biphenyl analysis

Samples were analyzed for seven indicator PCBs: PCB28, PCB52, PCB101, PCB118 (a dioxin like PCB), PCB138, PCB153 and PCB180. PCB extraction and clean-up was performed on 5 mL of serum spiked with 5 ng of each ^13^C_12_-labelled PCB (Wellington Laboratories) in 50 mL Falcon tubes using a previously described protocol (31). In brief, samples were vortexed for 1 minute, left to stand for 30 minutes and manually shaken for 1 minute with 6 mL acetonitrile, 3 mL milliQ, 5 g anhydrous MgSO_4_, 1 g NaCl and a ceramic homogenizer. Samples were centrifuged at 4500 RPM for 8 minutes at 10°C and the supernatant transferred to a glass tube which was evaporated to near-dryness on a hot plate using a gentle stream of nitrogen. Samples were reconstituted in approximately 1 mL hexane and to each sample, 1 mL of >98% concentrated sulfuric acid was added prior to being vortexed for no less than 30 s. Following overnight separation at <4°C, the organic layer was transferred onto a preconditioned silica SPE cartridge (Supelco LC-Si, 3 mL/500 mg). Target compounds were eluted into a 15 mL glass tube using 6 mL hexane. Clean extracts were evaporated to near-dryness, reconstituted in 50 µL hexane containing 2.5 ng ^13^C_12_-PCB-141 as a recovery standard, and transferred to inserted autosampler vials prior to analysis. PCBs were determined using high resolution gas chromatography (Thermofisher TRACE 1300) coupled with high resolution mass spectrometry (HRGC/HRMS, Thermofisher DFS) with quality assurance checks using previously described methods (32). In brief, the injector was operated in splitless mode with separation achieved on an Agilent DB-5ms column (30 m length x 0.25 mm in diameter x 0.25 µm film thickness). Analyzes were conducted in multiple ion detection (MID) mode at 10,000 resolution (10% valley definition). The inlet was held at 250°C, and the transfer line and source at 280°C. The flow rate was 1.0 mL/min. The GC oven was held at 80°C for two minutes before ramping to 180°C at 20°C/min for 0.5 min and 300°C at 10°C/min for 5 minutes. For quality assurance purposes, a blank sample was extracted as every 6^th^ sample (n=10) alternating between 5 mL of MilliQ (reagent blank) and 5 mL bovine serum (field blank). As no blank samples contained target compounds at concentrations >5% of samples concentrations, no blank corrections occurred. Bovine serum (5 mL, n=5) fortified with target compounds were used to assess precision and accuracy. 30 μL of solution containing 0.2 ng/μL of all target compounds in methanol was added to each aliquot, which was then vortexed for 1 min and left at <4°C overnight. Samples were analyzed as real samples using the protocols described above. Average recoveries ranged between 80 - 120% with a relative standard deviation of <15%. The limit of reporting (LOR) for PCB28, PCB52 and PCB101 was 1 ng/g Lipid, and PCB118, PCB138, PCB153 and PCB180, 0.1, 0.15, 0.3 and 0.2 ng/g Lipid, respectively. A sum PCB (∑PCB) variable was calculated by adding the molar concentrations of PCB congeners analyzed.

### 2.5 Statistical analysis

No published studies are available upon which to base a power calculation that detail changes in miRNAs to PCBs. A publication by Birkett and Day (33) reviewed pilot study sample size; the authors stated that, at a minimum, 20 degrees-of-freedom is necessary for estimation of effect size and variability. To meet that criterion, we therefore needed to analyse serum samples from a minimum of 20 patients per cohort and therefore 29 participants per group were recruited to allow for dropouts. Descriptive data are presented as mean ± standard deviation (SD) for continuous data. Metabolic outcomes and hormone concentrations were assessed for normality and Independent T, or Mann-Whitney U tests were used to compare means/medians, as appropriate. As the study aimed to explore potential relationships and given the number of variables under examination with some missing values, we carried out correlation analysis. Potential correlations with miRNAs, PCBs and metabolic outcomes, and steroid and hormone levels were examined using exploratory Spearman’s rank order correlations due to non-normal distribution of the data. Correlations were carried out for the study cohort and stratified based on group, either healthy controls or PCOS cases. Values below the LOR were treated as missing values and analyses were carried out using pairwise deletion. Correlations between PCB28, PCB52 and PCB101 were not performed due to the low detection frequency of these PCBs in the study cohort; 25%, 7% and 44%, respectively. A *p*-value of <0.05 was considered as indicative of statistical significance. Statistical analysis was carried out using Jamovi (version 1.8).

### 2.6 MiRNA functional enrichment analysis

MiRNA Enrichment Analysis was undertaken with the Annotation Tool (miEAA) (34) to better understand their interaction in various processes. Using default settings, we carried out Over-representation Analysis (ORA) with those miRNAs that correlated with PCBs in the study. Using the same list of miRNAs but ranked based on Spearman coefficients from most negative significance to most positive, we carried out Gene Set Enrichment Analysis (GSEA) adapted for miRNA.

## 3 Results

### 3.1 Demographics, metabolic outcomes, and hormone levels

PCOS cases and healthy controls did not differ in age, BMI, insulin, HOMA-IR or TSL (**Table 1**). PCOS cases had higher FAI (3.1 ± 2.9 vs 1.7 ± 3.4, *p*=0.002), AMH (56 ± 14 vs 24 ± 14 ng/mL, *p*<0.001) and LH (14.9 ± 14.3 vs 6.0 ± 8.3 IU/L) compared to controls. FBG was lower in PCOS cases (4.5 ± 0.8 vs 4.8 ± 0.3 nmol/L, *p*=0.04). There was no difference between CRP, HbA1c, FSH, estradiol or progesterone.

**Table 1:** Demographics, metabolic and hormone endpoints for women with PCOS and controls.

	Control (n=29)		PCOS (n=29)		
	Mean	SD	Mean	SD	p
Age (Years)	33	4	31	5	0.10
BMI (kg/m^2^)	25.4	3.6	26.0	3.8	0.49
Insulin (µIU/ml)	7.6	4.1	8.1	4.7	0.94
HOMA-IR	1.7	1.0	2.0	1.6	0.96
FBG (nmol/L)	4.8	0.3	4.5	0.8	0.04
TSL (mg/dL)	573	106	583	134	0.77
CRP (mg/L)	2.5	2.3	2.7	2.6	0.99
HbA1c (mmol/mol)	30.7	6.2	32.0	3.3	0.72
FAI	1.7	3.4	3.1	2.9	0.002
AMH (ng/mL)	24	14	56	14	< 0.001
LH (IU/L)	6.02	8.3	14.9	14.3	0.002
FSH (IU/L)	3.3	2.2	4.1	2.3	0.22
Estradiol (pmol/L)	457	301	431	463	0.25
Progesterone (nmol/L)	15	15.4	7.1	9.1	0.09

Body mass index (BMI); homeostatic model assessment for insulin resistance (HOMA-IR); fasting blood glucose (FBG); total serum lipids (TSL); C-reactive protein (CRP); glycosylated hemoglobin A1c (HbA1c); free androgen index (FAI); anti-müllerian hormone (AMH); luteinizing hormone (LH); follicle stimulating hormone (FSH); polycystic ovary syndrome (PCOS); standard deviation (SD); sample size (n); significance at p=0.05 Independent T or Mann-Whitney U (p).

### 3.2 Serum PCB levels

Geometric mean (GM) concentrations of frequently detected PCBs and the ∑PCB variable did not differ between PCOS cases and healthy controls (*p*=ns) (**Table 2**), as was previously reported in this study cohort (35).

**Table 2:** Serum PCB concentrations in controls and PCOS cases (35).

	LOR (ng/g Lipid)	Control (n=29)		PCOS (n=29)		
		GM (95% CI)	n (%)	GM (95% CI)	n (%)	p
PCB118	0.1	5.1 (4.6 - 5.8)	29 (100)	5.4 (4.7 - 6.2)	29 (100)	0.84
PCB138	0.15	11.6 (10.0 - 13.5)	29 (100)	10.1 (8.4 - 12.1)	29 (100)	0.11
PCB153	0.3	15 (12.6 – 18.0)	29 (100)	12.2 (9.8 - 15.3)	29 (100)	0.09
PCB180	0.2	13.5 (11.5 - 15.9)	29 (100)	10.7 (8.8 – 13.0)	29 (100)	0.08
ΣPCB		48.3 (42.1 – 55.5)	29 (100)	41.7 (34.9 – 49.8)	29 (100)	0.11

Limit of reporting (LOR); polycystic ovarian syndrome (PCOS); geometric mean (GM); confidence interval (CI); sample size (n); detection frequency (%); significance at p=0.05 Mann-Whitney U (p).

### 3.3 Serum miRNA levels

A total of 179 miRNAs were detected in serum samples from both PCOS cases and healthy controls (21). 120 miRNAs had a detection frequency of 100%, 28 miRNAs had a detection frequency of 98%, 7 miRNAs had a detection frequency of 97% and 23 miRNAs had a detection frequency of 71-95%. Hsa-miR-208a-3p had <14% detection frequency and was not included in the analysis (**Supplementary Table S1**). All 178 miRNAs analysed were common amongst PCOS subjects and controls.

### 3.4 Serum miRNA correlations with serum PCB levels in study cohort, and separately in healthy controls and PCOS cases

Exploratory Spearman’s rank order correlations of the most frequently detected PCBs (PCB118, PCB138, PCB153, PCB180) and ∑PCB were examined with miRNAs. In the total combined study cohort, hsa-miR-139-5p correlated with increasing concentration of PCB118 (ρ=0.30, *p*=0.03); hsa-miR-424-5p negatively correlated with PCB138, PCB153, PCB180, and ∑PCB (ρ>-0.29, *p*<0.05); hsa-miR-195-5p negatively correlated with PCB138 (ρ=-0.30, *p*=0.03); hsa-miR-335-5p negatively correlated with PCB138, PCB153, and ∑PCB (ρ>-0.27, *p*<0.05) (**Table 3**). No other significant correlations were found between PCBs and miRNAs in the combined study cohort. When stratified, a greater number of miRNAs correlated with PCBs in healthy controls than PCOS (25 versus 1) (**Table 4**).

**Table 3:** Exploratory Spearman ρ coefficients for PCB concentrations and miRNAs, in study cohort (n=58).

	PCB118		PCB138		PCB153		PCB180		∑PCBs	
	(ng/g Lipid)		(ng/g Lipid)		(ng/g Lipid)		(ng/g Lipid)		(ng/g Lipid)	
hsa-miR-139-5p	0.3	*	0.1		0.09		0.11		0.07	
hsa-miR-424-5p	-0.16		-0.31	*	-0.3	*	-0.29	*	-0.32	*
hsa-miR-195-5p	-0.13		-0.3	*	-0.27		-0.25		-0.27	
hsa-miR-335-5p	-0.17		-0.29	*	-0.27	*	-0.22		-0.32	*

Polychlorinated biphenyl (PCB); sum of PCBs (∑PCBs); p < 0.05 (*).

**Table 4:** Exploratory Spearman ρ coefficients for PCB concentrations and miRNAs, in controls and PCOS cases (n=29), showing that 25 miRNAs in controls correlated with PCBs but only one miRNA correlated with PCBs in PCOS.

	PCB118	PCB138	PCB153	PCB180	∑PCBs	Association with menstrual cycle/reproductive system
Controls						
hsa-miR-339-3p	-0.01	-0.17	-0.22	-0.38*	-0.27	Identified via GEO analysis to be upregulated and differential expressed in early and late luteal phases of the menstrual cycle in those with endometriosis compared to controls (36)
hsa-miR-154-5p	0.51*	0.19	0.23	-0.03	0.18	Potential biomarker for endometrial function in endometriosis (37)
hsa-miR-27b-3p	-0.14	-0.34	-0.36	-0.41*	-0.39*	E2 downregulates miR-27b thereby leading to upregulation of genes important for vascularization and angiogenesis of the endometrium during the menstrual cycle and decidualization (38)
hsa-miR-374b-5p	-0.27	-0.42*	-0.41*	-0.43*	-0.44*	Significantly down regulated in women with low versus normal ovarian reserve and correlated to AMH (39)
hsa-miR-23b-3p	-0.07	-0.28	-0.29	-0.41*	-0.35	Predicted to be important in female infertility by protein-protein analysis (40)
*hsa-miR-26a-5p*	-0.2	-0.35	-0.35	-0.41*	-0.38*	Positive correlation with LH in this study
hsa-let-7e-5p	-0.18	-0.42*	-0.35	-0.39*	-0.35	Suggested important role in ovarian function (41)
***hsa-miR-139-5p***	0.39*	0.13	0.17	0.01	0.13	Association of miR-139 with premature ovarian failure (41). Negative correlation with AMH in this study
hsa-miR-28-5p	-0.11	-0.25	-0.3	-0.44*	-0.32	Possible link with endometriosis (42). Significantly down regulated in women with high versus normal ovarian reserve and correlated to AMH (39)
*hsa-miR-99b-5p*	-0.15	-0.38*	-0.38*	-0.33	-0.36	Positive correlation with estradiol in this study
hsa-miR-326	-0.12	-0.25	-0.28	-0.4*	-0.35	miR-326 down-regulates *CYP19A1* expression and estradiol-17b production in an animal model (43). Menstrual cycle specific regulation with downregulation in secretory phase in healthy women which was lost in women with endometriosis (37)
hsa-miR-146a-5p	-0.17	-0.37	-0.38*	-0.43*	-0.41*	Upregulated in premature ovarian failure (44)
Controls						
**hsa-miR-424-5p**	-0.1	-0.39*	-0.32	-0.35	-0.34	Upregulated in the endometrium of repeated implantation failure patients and targets SSPI which is associated with infertility (45)
*hsa-miR-146b-5p*	-0.12	-0.34	-0.3	-0.41*	-0.35	Association of miR-146 with premature ovarian failure (41). Significantly down regulated in women with low versus normal ovarian reserve and correlated to AMH (39). Negative correlation with AMH and positive correlation with estradiol in this study
hsa-miR-874-3p	-0.09	-0.42*	-0.31	-0.34	-0.36	Upregulated in the secretory endometrium of repeated implantation failure patients compared with fertile controls (46)
*hsa-miR-193a-5p*	-0.22	-0.38*	-0.33	-0.23	-0.34	Positive correlation with progesterone and LH in this study
*hsa-miR-2110*	-0.08	-0.26	-0.24	-0.37*	-0.28	Positive correlation with LH in this study
hsa-miR-331-3p	-0.1	-0.33	-0.31	-0.39*	-0.33	Upregulated in the endometrial receptive phase (47)
hsa-miR-199a-5p	-0.05	-0.22	-0.28	-0.38*	-0.31	Significant increased expression in women with endometriosis (48)
***hsa-miR-195-5p***	-0.28	-0.47*	-0.46*	-0.32	-0.42*	Positive correlation with LH and FSH in this study
hsa-miR-151a-3p	-0.08	-0.36	-0.36	-0.43*	-0.37	Association of miR-151 with premature ovarian failure (41)
***hsa-miR-335-5p***	-0.3	-0.52**	-0.4*	-0.37*	-0.43*	Suppresses granulosa cell proliferation through potential mTOR pathway via inhibition of *SGK3* expression (49). Positive correlation with FSH in entire study cohort
hsa-miR-199a-3p	-0.11	-0.34	-0.36	-0.45*	-0.4*	Suggested important role of miR-199 in ovarian function based on preferential expression in the ovaries (41)
hsa-miR-335-3p	-0.25	-0.41	-0.38	-0.5*	-0.43*	Down regulated in minimal-mild endometriosis patients compared to controls (50)
hsa-miR-21-5p	-0.14	-0.37*	-0.29	-0.27	-0.31	Possible link with endometriosis (42). Significantly down regulated in women with low versus normal ovarian reserve and significantly correlated to AMH (39)
PCOS						
*hsa-miR-193a-5p*	0.25	0.36	0.38*	0.43	0.33	miR-193 involved with progesterone regulation (41). Positive correlation with estradiol in this study

The four miRNAs in bold are those associated with PCBs for the entire cohort (n=58). The miRNAs in italics are those that correlated with parameters of the menstrual cycle. Association of each of the miRNAs correlating with PCBs and evidence of their association with the menstrual cycle/reproductive system is reported.

Polychlorinated biphenyl (PCB); sum of PCBs (∑PCBs); polycystic ovary syndrome (PCOS); p < .05 (*); p < .01 (**).

### 3.5 Serum miRNA correlations with metabolic outcomes and hormone levels in study cohort, and separately in health controls and PCOS cases

We examined correlations between miRNAs and metabolic and hormone parameters for miRNAs that correlated with PCBs in the entire study cohort (4 miRNAs), in PCOS cases (1 miRNA) and healthy controls (25 miRNAs). No correlations were observed between the 4 miRNAs that correlated with PCBs in the entire study cohort for metabolic outcomes or hormone levels for BMI, insulin, insulin resistance (HOMA-IR), TSL, inflammation (CRP) or HbA1c (**Supplementary Table S2**). The only correlation found was between hsa-miR-335-5p and FSH (ρ=0.294, *p*= 0.04) in the study cohort.

**Figure 1:**
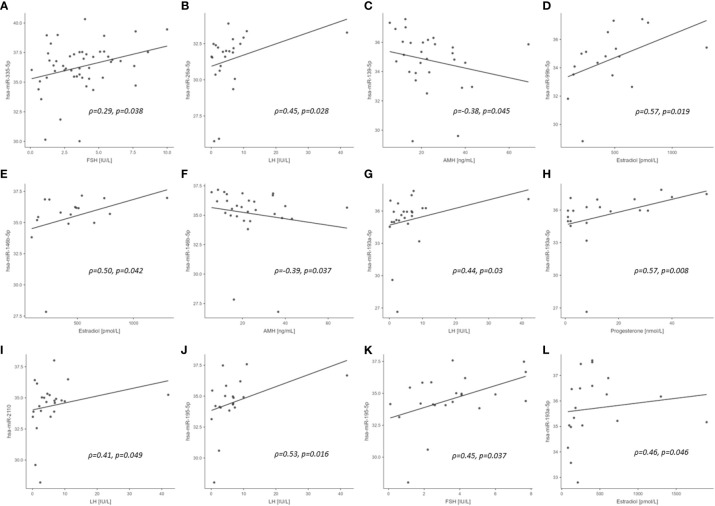
Scatterplots of PCB associated miRNA versus reproductive hormone concentrations in study cohort (significant correlations only). **(A)** entire cohort; **(B–K)**, control cohort; **(L)**, PCOS cohort. ρ, Spearman rho; *p*, significance.

No correlations were found for those miRNAs that correlated with PCBs for BMI, insulin, HOMA-IR and CRP in healthy control subjects or PCOS cases (n=29) (**Supplementary Table S3**). In control women, hsa-miR-26a-5p, hsa-miR-193a-5p, hsa-miR-2110 and hsa-miR-195-5p correlated with LH, hsa-miR-99b-5p and hsa-miR-146b-5p correlated with estradiol, hsa-miR-139-5p and hsa-miR-146b-5p correlated with AMH, hsa-miR-193a-5p correlated with progesterone, and hsa-miR-195-5p correlated with FSH (all *p*<0.05). In PCOS cases, hsa-miR-193a-5p correlated with estradiol. The significant exploratory Spearman rank order correlations for the entire cohort, control cohort alone (25 miRNAs correlating with PCBs), the PCOS cohort alone (one miRNA correlating with PCBs), and the association of those miRNAs with the menstrual cycle/reproductive system reported in the literature are shown in **Figure 1** and **Table 4**, respectively.

### 3.6 MiRNA functional enrichment analysis

MiRNA Enrichment Analysis and Annotation Tool (miEAA) (34) showed that the top five overrepresented diseases included cerebral hemorrhage traumatic, thyroid carcinoma, cardiovascular disease, dysautonomia familial and prostatic neoplasms; gene ontology included negative regulation of cholesterol transport, cardiac vascular smooth muscle cell development, coronary vein morphogenesis and cardiac septum morphogenesis; target genes included NFKB1, FASN, AURKAIP1, PTGS2, and SOCS6; pathways included adipocytokine signaling pathway, D-L1 expression and PD-1 checkpoint pathway in cancer, Type II diabetes mellitus, acute myeloid leukemia and inflammatory bowel disease.

Gene Set Enrichment Analysis (GSEA) adapted for the miRNA identified using default settings, revealed interactions with immune cells and sperm specific tissue.

## 4 Discussion

In this study, PCBs correlated with miRNAs; however, these correlations mostly occurred in the healthy control subjects rather than women with PCOS. Interestingly, most of the miRNAs identified were associated with hormones of the menstrual cycle or have been reported to have an association with female reproduction (**Table 4**). It is perhaps not surprising that so few PCBs correlated with miRNAs in the PCOS women, as these subjects were anovulatory. Therefore, the hormonal changes in the normal menstrual cycle that would be reflected in miRNA changes would be absent in PCOS women and, indeed, that appeared to be the case. It can thus be surmised that the deleterious effect of PCBs may be upon the hypothalamo-ovarian axis through effects on miRNAs, thus potentially compromising fertility.

The potential association of PCB exposure and fertility is controversial. In follicular fluid, inverse associations have been reported for PCB180 and antral follicular count, PCB138 and PCB153 and peak estradiol, and ΣPCBs and endometrial thickness (51). Conversely, in serum, only when PCB187 (heptachloro) with a half-life of 10.3 years (4) was considered as a mixture of lipophilic pollutants was an inverse association observed with follicle density (52). In this study, PCB153 and PCB180, both highly chlorinated with long half-lives, correlated with hsa-miR-374b-5p and hsa-miR-146a-5p that have been associated with ovarian function (39, 44). In addition, the dioxin-like PCB118 was associated with hsa-miR-139-5p which has been shown to be linked with premature ovarian failure (41). Higher levels of PCBs have also been reported to be associated with miscarriage (53) and here several miRNAs that have been associated with endometrial function also correlated with specific PCB congeners; miR-21-5p, hsa-miR-154-5p and hsa-miR-424-5p (37, 42, 45), correlated with PCB118 or PCB138, and hsa-miR-27b-3p and hsa-miR-331-3p (38, 47) correlated with PCB180. These results suggest that PCB118, PCB138 and PCB180 may be specifically involved in endometrial dysfunction via miRNA effects.

PCBs have been associated with endometriosis, which causes pelvic pain and infertility (54), a condition that is hormone-dependent due to an imbalance of progesterone and estrogen, a hormonal balance that may be affected by miRNAs, akin to the miRNAs associated with the menstrual hormones in this report. In this study, six PCB-associated miRNAs were reported to be linked to endometriosis: hsa-miR-339-3p, hsa-miR-28-5p, hsa-miR-199a-5p, hsa-miR-335-3p (correlated with PCB180), hsa-miR-154-5p (correlated with PCB118), and hsa-miR-21-5p (correlated with PCB138) (36, 37, 42, 48, 50). Of note, hsa-miR-154-5p has been identified as a potential biomarker of endometrial function in endometriosis (37). Conversely, in a large cross-sectional study of self-reported outcomes, whilst higher total PCB levels associated with fewer lifetime pregnancies, they did not correlate with the prevalence of infertility and pregnancy outcomes (55).

One novel observation in this study was that several of the miRNAs specifically correlated to the serum hormones of the hypothalamo-ovarian axis. These included hsa-miR-335-5p (associated with PCB153) with FSH; hsa-miR-26a-5p and hsa-miR-2110 (associated with PCB180) with LH; hsa-miR-99b-5p (associated with PCB138, PCB153 and PCB180), hsa-miR-146b-5p (associated with PCB180) and hsa-miR-193a-5p (associated with PCB153 with estradiol; hsa-miR-193a-5p (associated with PCB138) with progesterone and LH; hsa-miR-195-5p (associated with PCB138 and PCB153) with LH and FSH. Two of the miRNAs, hsa-miR-139-5p (associated with PCB118) and hsa-miR-146b-5p (associated with PCB180), correlated negatively with AMH indicating their association with reproduction. These negative correlations could perhaps have been anticipated for hsa-miR-139-5p and hsa-miR-146 that have been associated with premature ovarian failure (41). These PCB/miRNA/hormone associations strongly suggest that the highly chlorinated congeners, those with high lipophilicity and low biotransformation rates (PCB138, PCB153 and PCB180) may modulate miRNAs associated with reproductive hormonal levels. However, the level of the effect is unclear. Conversely, the dioxin-like PCB118 appears not to be associated with the reproductive hormones LH, FSH, progesterone and estradiol in the study cohort.

If there were to be an association between any of the non-reproductive hormones, metabolic parameters, and the PCBs between PCOS and healthy control women, it would have manifest with the highly chlorinated congeners (PCB118, PCB138, PCB153 and PCB180) that have very long half lives in the body. What was surprising was that the PCB associated miRNAs appeared to be restricted to the hormones associated with the menstrual cycle in control women more so than in PCOS cases. PCBs are lipophilic and therefore may accumulate in adipose tissue and therefore are associated with BMI (56). Only in this study design, where women were similar for obesity and age and then evaluated for differences between PCOS and non-PCOS cohorts, could removal of confounding factors achieve an adequate answer.

MiRNA Enrichment Analysis showed interactions with cardiovascular disease (57), regulation of cholesterol transport (and association with dyslipidemia (58)) and type 2 diabetes (58) all of which are associated with PCOS, there were other interactions that appeared unrelated to PCOS such as thyroid carcinoma. Gene Set Enrichment Analysis was less revealing but showed interactions with immune cells that are known to be dysfunctional in PCOS (59). However, due to the inconsistent trends in ORA and GSEA analysis, we focused on the published literature of individual miRNAs.

We previously reported that PCBs did not differ in this cohort (35). Why the PCBs did not differ between the healthy controls and PCOS cases could perhaps be attributed to the study design of similar age and BMI Caucasian women that were from the same geographical area in northern England which was within a 20-mile radius from the IVF center under study. The strengths of this study lie in the study design of comparable non-obese PCOS subjects and healthy controls with similar age and BMI, the measurement of both metabolic parameters and hormone levels, and the representation of a potential sensitive subpopulation to exogenous exposure to endocrine disruptors and their effects on circulating miRNA levels. The limitations of the study include the small sample size, the limited number of PCBs measured, the lack of data on potential PCB exposure sources in the study cohort, including occupation and education, and the potential lack of generalizability to ethnicities other than a Caucasian population. Given the exploratory nature of the analysis, lack of correction for multiple testing and the small sample size, there are limitations regarding the potential correlations with some exhibiting only weak correlations and with outliers present. In studies where PCBs are significantly different between groups, which was not the case in this study, it is possible that results may differ. However, this study has the potential to inform future epidemiological studies of miRNA expression with exogenous exposure to endocrine disruptors on a larger cohort of women. This study further highlights the need for such populations to be similar for both age and BMI to ensure studies are comparable.

## 5 Conclusion

In conclusion, in this cohort of women, with no difference in age and BMI, and with similar PCB levels, the miRNAs correlating to the PCBs associated with menstrual cycle factors in control women versus PCOS women. The lack of association in PCOS women may be a result of their anovulatory cycle. The PCB-associated miRNAs did not correlate with non-reproductive hormonal and metabolic parameters. Overall, this suggests that PCB effects on miRNA may result in changes to the hypothalamo-ovarian axis that may thus affect fertility through modulation of miRNA affecting ovarian reserve, endometriosis, endometrial function, and effects on the hormones of the hypothalamo-ovarian axis. Further studies on a larger cohort are required to expound the results presented here and whether PCB effects on miRNA impact the etiology and pathophysiology of PCOS.

## Data availability statement

The raw data supporting the conclusions of this article will be made available by the authors, without undue reservation.

## Ethics statement

The studies involving humans were approved by The Yorkshire and The Humber NRES ethical committee, UK (approval number 02/03/043). The studies were conducted in accordance with the local legislation and institutional requirements. The participants provided their written informed consent to participate in this study.

## Author contributions

EB: visualization, formal analysis, writing—original draft preparation, writing—review and editing. AB: writing—original draft preparation, writing—review and editing. DD: investigation, writing—review and editing. TS: Conceptualization, methodology. SA: Conceptualization, methodology, validation, resources, data curation, visualization, writing—review and editing. All authors have read and agreed to the published version of the manuscript.
