# Supplementary material for: Serum polychlorinated biphenyl levels and circulating miRNAs in non-obese women with and without polycystic ovary syndrome
Source: Front Endocrinol (Lausanne). 2023 Sep 18;14:1233484. doi: 10.3389/fendo.2023.1233484 (PMC10544902; doi:10.3389/fendo.2023.1233484)
Supplement: Supplementary file 1 [file DataSheet_1.docx]

Supplementary Material

Serum polychlorinated biphenyl levels and circulating miRNAs in non-obese women with and without polycystic ovary syndrome

Edwina Brennan^1,*^, Alexandra Butler^1^, Daniel S. Drage^2,3^, Thozhukat Sathyapalan^4^, Stephen L. Atkin^1^

*** Correspondence:** Edwina Brennan: [ebrennan@rcsi.com](mailto:ebrennan@rcsi.com)

# Supplementary Figures

**Supplementary Figure S1**. Flow chart of participant recruitment.


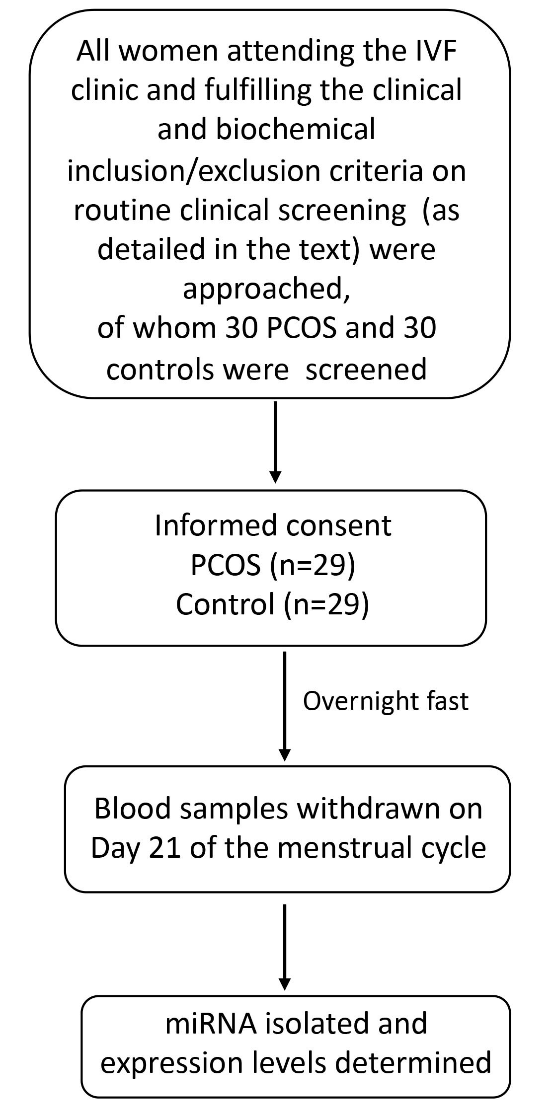


# Supplementary Tables

**Supplementary Table S1.** miRNA in PCOS cases and controls.

| miRNA | n | % | miRNA | n | % | miRNA | n | % |
| --- | --- | --- | --- | --- | --- | --- | --- | --- |
| hsa-miR-1260a | 58 | 100 | hsa-miR-141-3p | 54 | 93 | hsa-miR-150-5p | 58 | 100 |
| hsa-miR-133a-3p | 54 | 93 | hsa-let-7a-5p | 58 | 100 | hsa-miR-222-3p | 56 | 97 |
| hsa-miR-144-5p | 56 | 97 | hsa-miR-766-3p | 57 | 98 | hsa-miR-20b-5p | 58 | 100 |
| hsa-miR-205-5p | 57 | 98 | hsa-miR-23b-3p | 58 | 100 | hsa-miR-26b-5p | 58 | 100 |
| hsa-miR-154-5p | 47 | 81 | hsa-miR-335-5p | 58 | 100 | hsa-miR-194-5p | 58 | 100 |
| hsa-miR-874-3p | 58 | 100 | hsa-miR-23a-3p | 58 | 100 | hsa-miR-130a-3p | 57 | 98 |
| hsa-miR-136-3p | 51 | 88 | hsa-miR-99a-5p | 57 | 98 | hsa-miR-590-5p | 58 | 100 |
| hsa-miR-375 | 57 | 98 | hsa-miR-133b | 51 | 88 | hsa-miR-151a-5p | 58 | 100 |
| hsa-miR-424-5p | 58 | 100 | hsa-miR-877-5p | 55 | 95 | hsa-miR-376a-3p | 55 | 95 |
| hsa-miR-28-5p | 50 | 86 | hsa-miR-505-3p | 58 | 100 | hsa-miR-140-3p | 58 | 100 |
| hsa-miR-18b-5p | 58 | 100 | hsa-miR-181a-5p | 58 | 100 | hsa-miR-139-5p | 52 | 90 |
| hsa-miR-10b-5p | 58 | 100 | hsa-miR-223-5p | 56 | 97 | hsa-miR-126-5p | 58 | 100 |
| hsa-miR-328-3p | 58 | 100 | hsa-miR-33a-5p | 58 | 100 | hsa-miR-532-3p | 57 | 98 |
| hsa-miR-2110 | 58 | 100 | hsa-miR-210-3p | 58 | 100 | hsa-miR-185-5p | 57 | 98 |
| hsa-miR-339-3p | 58 | 100 | hsa-miR-132-3p | 58 | 100 | hsa-miR-376c-3p | 56 | 97 |
| hsa-let-7b-3p | 57 | 98 | hsa-miR-16-2-3p | 57 | 98 | hsa-miR-22-3p | 58 | 100 |
| hsa-miR-100-5p | 57 | 98 | hsa-let-7e-5p | 58 | 100 | hsa-miR-92b-3p | 55 | 95 |
| hsa-miR-409-3p | 53 | 91 | hsa-miR-17-5p | 57 | 98 | hsa-miR-361-5p | 57 | 98 |
| hsa-miR-497-5p | 58 | 100 | hsa-let-7d-3p | 58 | 100 | hsa-miR-532-5p | 58 | 100 |
| hsa-miR-501-3p | 57 | 98 | hsa-miR-301a-3p | 58 | 100 | hsa-miR-374a-5p | 58 | 100 |
| hsa-let-7c-5p | 58 | 100 | hsa-miR-26a-5p | 58 | 100 | hsa-miR-197-3p | 58 | 100 |
| hsa-miR-192-5p | 58 | 100 | hsa-miR-342-3p | 58 | 100 | hsa-miR-338-3p | 58 | 100 |
| hsa-miR-362-3p | 58 | 100 | hsa-miR-144-3p | 58 | 100 | hsa-miR-223-3p | 58 | 100 |
| hsa-miR-140-5p | 58 | 100 | hsa-miR-34a-5p | 57 | 98 | hsa-miR-148a-3p | 58 | 100 |
| hsa-miR-136-5p | 57 | 98 | hsa-miR-365a-3p | 58 | 100 | hsa-miR-30e-5p | 57 | 98 |
| hsa-let-7d-5p | 58 | 100 | hsa-miR-485-3p | 46 | 79 | hsa-miR-24-3p | 58 | 100 |
| hsa-miR-423-3p | 58 | 100 | hsa-miR-15b-5p | 58 | 100 | hsa-miR-103a-3p | 57 | 98 |
| hsa-miR-195-5p | 53 | 91 | hsa-miR-152-3p | 56 | 97 | hsa-miR-29a-3p | 58 | 100 |
| hsa-miR-106b-3p | 55 | 95 | hsa-miR-29b-3p | 58 | 100 | hsa-miR-93-3p | 57 | 98 |
| hsa-miR-425-3p | 58 | 100 | hsa-miR-885-5p | 55 | 95 | hsa-miR-326 | 58 | 100 |
| hsa-let-7b-5p | 58 | 100 | hsa-miR-30b-5p | 57 | 98 | hsa-miR-107 | 58 | 100 |
| hsa-miR-324-5p | 58 | 100 | hsa-miR-145-5p | 58 | 100 | hsa-miR-30d-5p | 58 | 100 |
| hsa-miR-7-5p | 58 | 100 | hsa-miR-18a-5p | 58 | 100 | hsa-miR-423-5p | 58 | 100 |
| hsa-miR-125a-5p | 58 | 100 | hsa-miR-374b-5p | 58 | 100 | hsa-miR-200c-3p | 41 | 71 |
| hsa-miR-1 | 46 | 79 | hsa-miR-454-3p | 57 | 98 | hsa-miR-451a | 57 | 98 |

**Supplementary Table S1 (Cont.).** miRNA in PCOS cases and controls.

| miRNA | n | % | miRNA | n | % | miRNA | n | % |
| --- | --- | --- | --- | --- | --- | --- | --- | --- |
| hsa-miR-128-3p | 58 | 100 | hsa-miR-484 | 58 | 100 | hsa-miR-148b-3p | 58 | 100 |
| hsa-miR-130b-3p | 58 | 100 | hsa-miR-363-3p | 58 | 100 | hsa-miR-32-5p | 58 | 100 |
| hsa-miR-382-5p | 50 | 86 | hsa-miR-7-1-3p | 58 | 100 | hsa-let-7i-5p | 58 | 100 |
| hsa-miR-16-5p | 58 | 100 | hsa-miR-155-5p | 49 | 85 |  |  |  |
| hsa-miR-19a-3p | 58 | 100 | hsa-miR-142-3p | 58 | 100 |  |  |  |
| hsa-miR-151a-3p | 57 | 98 | hsa-miR-502-3p | 58 | 100 |  |  |  |
| hsa-miR-143-3p | 58 | 100 | hsa-miR-27a-3p | 58 | 100 |  |  |  |
| hsa-miR-193a-5p | 58 | 100 | hsa-miR-320b | 58 | 100 |  |  |  |
| hsa-miR-92a-3p | 58 | 100 | hsa-miR-324-3p | 58 | 100 |  |  |  |
| hsa-miR-125b-5p | 58 | 100 | hsa-miR-495-3p | 56 | 97 |  |  |  |
| hsa-miR-122-5p | 58 | 100 | hsa-miR-320c | 58 | 100 |  |  |  |
| hsa-miR-191-5p | 58 | 100 | hsa-miR-19b-3p | 58 | 100 |  |  |  |
| hsa-miR-28-3p | 58 | 100 | mmu-miR-378a-3p | 58 | 100 |  |  |  |
| hsa-miR-574-3p | 57 | 98 | hsa-miR-199a-3p | 58 | 100 |  |  |  |
| hsa-miR-27b-3p | 58 | 100 | hsa-miR-660-5p | 58 | 100 |  |  |  |
| hsa-miR-215-5p | 58 | 100 | hsa-miR-421 | 58 | 100 |  |  |  |
| hsa-miR-320a | 57 | 98 | hsa-miR-20a-5p | 58 | 100 |  |  |  |
| hsa-miR-127-3p | 50 | 86 | hsa-miR-30e-3p | 56 | 97 |  |  |  |
| hsa-miR-126-3p | 58 | 100 | hsa-miR-22-5p | 58 | 100 |  |  |  |
| hsa-miR-584-5p | 58 | 100 | hsa-miR-106a-5p | 57 | 98 |  |  |  |
| hsa-miR-320d | 58 | 100 | hsa-miR-339-5p | 58 | 100 |  |  |  |
| hsa-miR-335-3p | 45 | 78 | hsa-miR-93-5p | 58 | 100 |  |  |  |
| hsa-miR-331-3p | 57 | 98 | hsa-miR-146b-5p | 58 | 100 |  |  |  |
| hsa-miR-186-5p | 58 | 100 | hsa-miR-15b-3p | 58 | 100 |  |  |  |
| hsa-miR-486-5p | 58 | 100 | hsa-miR-146a-5p | 57 | 98 |  |  |  |
| hsa-miR-142-5p | 58 | 100 | hsa-miR-29c-3p | 58 | 100 |  |  |  |
| hsa-let-7g-5p | 58 | 100 | hsa-let-7f-5p | 58 | 100 |  |  |  |
| hsa-miR-221-3p | 57 | 98 | hsa-miR-106b-5p | 58 | 100 |  |  |  |
| hsa-miR-101-3p | 58 | 100 | hsa-miR-15a-5p | 58 | 100 |  |  |  |
| hsa-miR-199a-5p | 58 | 100 | hsa-miR-425-5p | 58 | 100 |  |  |  |
| hsa-miR-30a-5p | 58 | 100 | hsa-miR-629-5p | 58 | 100 |  |  |  |
| hsa-miR-30c-5p | 58 | 100 | hsa-miR-543 | 43 | 74 |  |  |  |
| hsa-miR-21-5p | 58 | 100 | hsa-miR-25-3p | 58 | 100 |  |  |  |
| hsa-miR-652-3p | 58 | 100 | hsa-miR-483-5p | 58 | 100 |  |  |  |
| hsa-miR-99b-5p | 58 | 100 | hsa-miR-200a-3p | 45 | 78 |  |  |  |

**Supplementary Table S2.** Insignificant exploratory Spearman ρ coefficients for miRNA that correlated with polychlorinated biphenyls and body mass index (BMI), insulin, insulin resistance (HOMA-IR), inflammation (CRP), total serum lipids (TSL) and HbA1c in study cohort (n = 58).

|  | BMI (kg/m^2^) | Insulin (μIU/mL) | HOMA-IR | CRP (mg/L) | TSL  (mg/dL) | HbA1c  (mmol/mol) |
| --- | --- | --- | --- | --- | --- | --- |
| hsa-miR-139-5p | -0.09 | -0.12 | -0.17 | -0.16 | -0.21 | -0.22 |
| hsa-miR-424-5p | 0.06 | -0.08 | -0.07 | 0.21 | 0.03 | 0.06 |
| hsa-miR-195-5p | 0.11 | 0.10 | 0.14 | 0.20 | -0.09 | -0.06 |
| hsa-miR-335-5p | 0.23 | 0.21 | 0.25 | 0.04 | -0.10 | 0.09 |

body mass index (BMI); homeostatic model assessment for insulin resistance (HOMA-IR);

C reactive protein (CRP); total serum lipids (TSL); glycosylated hemoglobin A1c (HbA1c)

**Supplementary Table S3.** Insignificant exploratory Spearman ρ coefficients for miRNA that correlated with polychlorinated biphenyls and Body Mass Index, insulin and insulin resistance (HOMA-IR) and inflammation (CRP) in control subjects (n=29) and PCOS cases (n=29).

|  | BMI (kg/m^2^) | Insulin (μIU/mL) | HOMA-IR | CRP (mg/L) |
| --- | --- | --- | --- | --- |
| Controls | | | | |
| hsa-miR-339-3p | 0.14 | 0.17 | 0.13 | -0.20 |
| hsa-miR-154-5p | -0.08 | -0.17 | -0.12 | -0.34 |
| hsa-miR-27b-3p | 0.10 | -0.04 | -0.06 | -0.07 |
| hsa-miR-374b-5p | 0.34 | 0.18 | 0.15 | -0.04 |
| hsa-miR-23b-3p | 0.09 | -0.01 | -0.02 | -0.17 |
| hsa-miR-26a-5p | 0.17 | 0.13 | 0.10 | 0.06 |
| hsa-let-7e-5p | 0.17 | 0.16 | 0.15 | 0.14 |
| hsa-miR-139-5p | 0.07 | -0.15 | -0.14 | -0.25 |
| hsa-miR-28-5p | -0.07 | -0.05 | -0.03 | -0.21 |
| hsa-miR-99b-5p | 0.13 | 0.14 | 0.13 | -0.05 |
| hsa-miR-326 | 0.11 | 0.09 | 0.05 | -0.24 |
| hsa-miR-146a-5p | 0.13 | 0.04 | 0.02 | -0.07 |
| hsa-miR-424-5p | 0.16 | 0.02 | -0.01 | 0.27 |
| hsa-miR-146b-5p | 0.06 | -0.09 | -0.12 | -0.13 |
| hsa-miR-874-3p | 0.32 | 0.26 | 0.22 | 0.01 |
| hsa-miR-193a-5p | 0.05 | 0.04 | -0.03 | 0.04 |
| hsa-miR-2110 | 0.12 | 0.05 | 0.00 | -0.11 |
| hsa-miR-331-3p | 0.06 | 0.00 | -0.04 | 0.02 |
| hsa-miR-199a-5p | 0.14 | 0.01 | -0.00 | -0.15 |
| hsa-miR-195-5p | 0.01 | 0.24 | 0.16 | 0.12 |
| hsa-miR-151a-3p | 0.05 | 0.07 | 0.04 | 0.01 |
| hsa-miR-335-5p | 0.21 | 0.28 | 0.25 | 0.01 |
| hsa-miR-199a-3p | 0.10 | -0.04 | -0.06 | -0.09 |
| hsa-miR-335-3p | -0.10 | 0.01 | -0.04 | 0.11 |
| hsa-miR-21-5p | 0.25 | 0.11 | 0.07 | 0.15 |
| PCOS | | | | |
| hsa-miR-193a-5p | -0.02 | -0.19 | -0.10 | 0.00 |

body mass index (BMI); homeostatic model assessment for insulin resistance (HOMA-IR); C reactive protein (CRP)
